# 2,6-Dimethyl­anilinium chloride monohydrate

**DOI:** 10.1107/S1600536808039159

**Published:** 2008-11-29

**Authors:** Wajda Smirani, Olfa Amri, Mohamed Rzaigui

**Affiliations:** a Laboratoire de Chimie des Matériaux, Faculté des Sciences de Bizerte, 7021 Zarzouna Bizerte, Tunisia

## Abstract

In the title hydrated mol­ecular salt, C_8_H_12_N^+^·Cl^−^·H_2_O, the component species inter­act by way of N—H⋯O, N—H⋯Cl and O—H⋯Cl hydrogen bonds, resulting in a three-dimensional network.

## Related literature

For related structures, see: Abid *et al.* (2007 ▶); Mrad *et al.* (2006 ▶). For hydrogen-bond motifs, see: Bernstein *et al.* (1995 ▶).
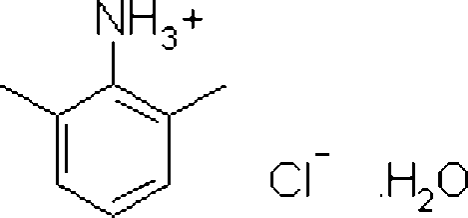


## Experimental

### 

#### Crystal data

C_8_H_12_N^+^·Cl^−^·H_2_O
*M* *_r_* = 175.65
Monoclinic, 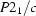

*a* = 8.676 (3) Å
*b* = 14.144 (3) Å
*c* = 7.913 (6) Å
β = 101.88 (5)°
*V* = 950.2 (8) Å^3^
*Z* = 4
Mo *K*α radiation
μ = 0.35 mm^−1^
*T* = 293 (2) K
0.20 × 0.13 × 0.10 mm

#### Data collection

Enraf–Nonius TurboCAD-4 diffractometer
Absorption correction: none
3722 measured reflections
2244 independent reflections
1827 reflections with *I* > 2σ(*I*)
*R*_int_ = 0.033
2 standard reflections frequency: 120 min intensity decay: 5%

#### Refinement

*R*[*F* ^2^ > 2σ(*F* ^2^)] = 0.031
*wR*(*F* ^2^) = 0.080
*S* = 1.04
2244 reflections
156 parameters
H-atom parameters not refined
Δρ_max_ = 0.17 e Å^−3^
Δρ_min_ = −0.24 e Å^−3^

### 

Data collection: *CAD-4 EXPRESS* (Enraf–Nonius, 1994 ▶); cell refinement: *CAD-4 EXPRESS*; data reduction: *XCAD4* (Harms & Wocadlo, 1995 ▶); program(s) used to solve structure: *SHELXS97* (Sheldrick, 2008 ▶); program(s) used to refine structure: *SHELXL97* (Sheldrick, 2008 ▶); molecular graphics: *ORTEP-3 for Windows* (Farrugia, 1997 ▶); software used to prepare material for publication: *WinGX* (Farrugia, 1999 ▶).

## Supplementary Material

Crystal structure: contains datablocks I, global. DOI: 10.1107/S1600536808039159/hb2860sup1.cif

Structure factors: contains datablocks I. DOI: 10.1107/S1600536808039159/hb2860Isup2.hkl

Additional supplementary materials:  crystallographic information; 3D view; checkCIF report

## Figures and Tables

**Table 1:** Hydrogen-bond geometry (Å, °)

*D*—H⋯*A*	*D*—H	H⋯*A*	*D*⋯*A*	*D*—H⋯*A*
O1—H1⋯Cl1	0.90 (2)	2.41 (2)	3.305 (3)	173 (2)
O1—H2⋯Cl1^i^	0.87 (3)	2.32 (3)	3.163 (3)	165 (2)
N1—H6⋯Cl1^ii^	0.893 (18)	2.392 (18)	3.235 (3)	157.5 (15)
N1—H7⋯O1	0.896 (16)	1.835 (16)	2.731 (3)	177.3 (17)
N1—H8⋯Cl1^iii^	0.883 (16)	2.414 (16)	3.265 (3)	162.8 (15)

Symmetry codes: (i) 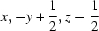; (ii) 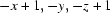; (iii) 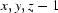.

**Fig. 1.**
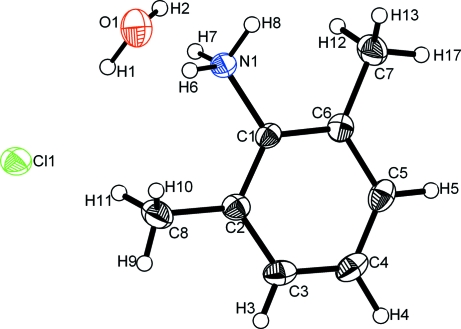
View of (I) with displacement ellipsoids for non-H atoms drawn at the 30% probability level (arbitrary spheres for the H atoms).

**Fig. 2.**
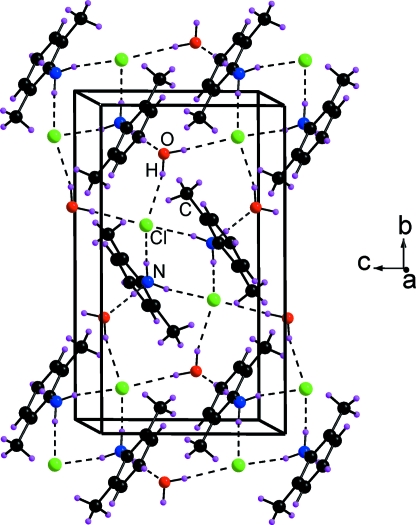
A perspective view of the packing in (I).

## supplementary crystallographic information

### Comment

As part of our ongoing studies of organic-inorganic hybrid networks containing the 2,6-xylidinium cation (Mrad *et al.*, 2006; Abid *et al.*, 2007) we now report the synthesis and structure of the title compound, (I).

As shown in Fig. 1, the asymmetric unit of (I) contains a 2,6-xylidinium cation, a chloride anion and a water molecule. A perspective view of the structure along the *a* axis is given in Fig. 2. It shows that two 2,6-xylidinium cations are interconnected through two chloride anions into dimers *via* two N—H···Cl bonds, characterized by N···Cl separations of 3.264 (3) and 3.235 (3) Å) and forming an 8-membered ring with graph-set *R*_2_^4^(8) (Bernstein *et al.*, 1995).

The title compound is a crystalline hydrate including one water of crystallization, which interconnect these dimers to each other to form layers parallel to the *(b, c)* plane, through N—H···O and O—H···Cl hydrogen bonds (Table 1).

Hydrogen bonds, electrostatic and van der Waals interactions participate to the cohesion of the three-dimensional network and add stability to this compound (Fig. 2). An examination of the organic group moiety geometrical features shows that the C—C and C—N bond lengths and the C—C—C and C—C—N angles are in the range usually found for this molecule (Abid *et al.*, 2007).

### Experimental

2,6-xylidinie and HCl were mixed in water in a 1: 1 molar ratio. The obtained solution was slowly evapored at room temperature to yield colourless blocks of (I).

### Refinement

The H atoms were located in a difference map and their positions and U_iso_ values were freely refined.

### Crystal data

**Table:**

C_8_H_12_N^+^·Cl^–^·H_2_O	*F*_000_ = 376
*M**_r_* = 175.65	*D*_x_ = 1.228 Mg m^−^^3^
Monoclinic, *P*2_1_/*c*	Mo *K*α radiation λ = 0.71073 Å
Hall symbol: -P 2ybc	Cell parameters from 25 reflections
*a* = 8.676 (3) Å	θ = 9.2–10.8º
*b* = 14.144 (3) Å	µ = 0.35 mm^−^^1^
*c* = 7.913 (6) Å	*T* = 293 (2) K
β = 101.88 (5)º	Block, colourless
*V* = 950.2 (8) Å^3^	0.20 × 0.13 × 0.10 mm
*Z* = 4	

### Data collection

**Table:**

Enraf–Nonius TurboCAD-4 diffractometer	θ_max_ = 28.0º
Monochromator: graphite	θ_min_ = 2.8º
*T* = 293 K	*h* = −5→11
Non–profiled ω scans	*k* = −18→0
Absorption correction: none	*l* = −10→10
3722 measured reflections	2 standard reflections
2244 independent reflections	every 120 min
1827 reflections with *I* > 2σ(*I*)	intensity decay: 5%
*R*_int_ = 0.033	

### Refinement

**Table:**

Refinement on *F*^2^	Secondary atom site location: difference Fourier map
Least-squares matrix: full	Hydrogen site location: inferred from neighbouring sites
*R*[*F*^2^ > 2σ(*F*^2^)] = 0.031	H-atom parameters not refined
*wR*(*F*^2^) = 0.080	*w* = 1/[σ^2^(*F*_o_^2^) + (0.0408*P*)^2^ + 0.1063*P*] where *P* = (*F*_o_^2^ + 2*F*_c_^2^)/3
*S* = 1.04	(Δ/σ)_max_ < 0.001
2244 reflections	Δρ_max_ = 0.17 e Å^−^^3^
156 parameters	Δρ_min_ = −0.24 e Å^−^^3^
Primary atom site location: structure-invariant direct methods	Extinction correction: none

### Special details

**Table:**

Geometry. All e.s.d.'s (except the e.s.d. in the dihedral angle between two l.s. planes) are estimated using the full covariance matrix. The cell e.s.d.'s are taken into account individually in the estimation of e.s.d.'s in distances, angles and torsion angles; correlations between e.s.d.'s in cell parameters are only used when they are defined by crystal symmetry. An approximate (isotropic) treatment of cell e.s.d.'s is used for estimating e.s.d.'s involving l.s. planes.
Refinement. Refinement of *F*^2^ against ALL reflections. The weighted *R*-factor *wR* and goodness of fit *S* are based on *F*^2^, conventional *R*-factors *R* are based on *F*, with *F* set to zero for negative *F*^2^. The threshold expression of *F*^2^ > σ(*F*^2^) is used only for calculating *R*-factors(gt) *etc*. and is not relevant to the choice of reflections for refinement. *R*-factors based on *F*^2^ are statistically about twice as large as those based on *F*, and *R*- factors based on ALL data will be even larger.

### Fractional atomic coordinates and isotropic or equivalent isotropic displacement parameters (Å^2^)

**Table:**

	*x*	*y*	*z*	*U*_iso_*/*U*_eq_	
H8	0.3326 (19)	0.0879 (11)	0.050 (2)	0.048 (4)*	
H7	0.3721 (19)	0.1013 (11)	0.233 (2)	0.045 (4)*	
H5	−0.159 (2)	0.2065 (13)	0.033 (2)	0.058 (4)*	
H6	0.3494 (19)	0.0085 (13)	0.161 (2)	0.053 (4)*	
H3	−0.121 (2)	−0.0291 (12)	0.318 (2)	0.055 (4)*	
H13	0.171 (2)	0.2037 (14)	−0.109 (2)	0.066 (5)*	
H11	0.265 (2)	−0.0493 (14)	0.427 (3)	0.070 (6)*	
H17	0.056 (2)	0.2698 (15)	−0.066 (2)	0.073 (5)*	
H2	0.488 (3)	0.2297 (19)	0.405 (3)	0.083 (7)*	
H4	−0.278 (2)	0.0943 (13)	0.191 (2)	0.067 (5)*	
H1	0.472 (3)	0.1562 (17)	0.518 (3)	0.090 (7)*	
H10	0.215 (2)	−0.1099 (13)	0.268 (2)	0.062 (5)*	
H9	0.124 (2)	−0.1045 (14)	0.406 (3)	0.071 (6)*	
H12	0.215 (3)	0.2560 (15)	0.065 (3)	0.087 (7)*	
N1	0.31170 (12)	0.06739 (8)	0.14878 (14)	0.0353 (2)	
C7	0.1308 (2)	0.22609 (11)	−0.0155 (2)	0.0510 (3)	
C8	0.1800 (2)	−0.07172 (12)	0.3454 (2)	0.0512 (3)	
C1	0.14475 (13)	0.07516 (8)	0.15925 (14)	0.0322 (2)	
C6	0.05793 (14)	0.15126 (9)	0.07822 (14)	0.0359 (3)	
C5	−0.09996 (16)	0.15637 (10)	0.08975 (17)	0.0435 (3)	
C2	0.08245 (14)	0.00745 (9)	0.25368 (15)	0.0367 (3)	
C3	−0.07586 (16)	0.01607 (10)	0.26089 (17)	0.0445 (3)	
C4	−0.16594 (15)	0.08936 (11)	0.18003 (18)	0.0466 (3)	
O1	0.48995 (14)	0.16863 (9)	0.41224 (15)	0.0580 (3)	
Cl1	0.45854 (4)	0.11499 (2)	0.81025 (4)	0.04716 (12)	

### Atomic displacement parameters (Å^2^)

**Table:**

	*U*^11^	*U*^22^	*U*^33^	*U*^12^	*U*^13^	*U*^23^
N1	0.0319 (5)	0.0398 (5)	0.0364 (5)	0.0052 (4)	0.0125 (4)	0.0025 (4)
C7	0.0553 (9)	0.0467 (7)	0.0546 (8)	0.0130 (7)	0.0200 (7)	0.0119 (6)
C8	0.0580 (9)	0.0523 (8)	0.0493 (8)	0.0100 (7)	0.0253 (7)	0.0123 (7)
C1	0.0282 (5)	0.0398 (6)	0.0296 (5)	0.0024 (4)	0.0083 (4)	−0.0058 (4)
C6	0.0370 (6)	0.0389 (6)	0.0318 (5)	0.0050 (5)	0.0074 (4)	−0.0049 (4)
C5	0.0356 (6)	0.0504 (7)	0.0428 (6)	0.0102 (6)	0.0039 (5)	−0.0091 (6)
C2	0.0378 (6)	0.0416 (6)	0.0328 (5)	0.0007 (5)	0.0121 (4)	−0.0047 (5)
C3	0.0402 (7)	0.0524 (8)	0.0452 (6)	−0.0068 (6)	0.0186 (5)	−0.0077 (6)
C4	0.0298 (6)	0.0602 (8)	0.0508 (7)	−0.0003 (5)	0.0107 (5)	−0.0145 (6)
O1	0.0654 (7)	0.0543 (7)	0.0509 (6)	0.0072 (5)	0.0039 (5)	−0.0041 (5)
Cl1	0.04621 (19)	0.04719 (19)	0.0534 (2)	0.01013 (14)	0.02267 (14)	0.00643 (13)

### Geometric parameters (Å, °)

**Table:**

N1—C1	1.4718 (16)	C1—C2	1.3907 (17)
N1—H8	0.883 (18)	C1—C6	1.3921 (17)
N1—H7	0.896 (17)	C6—C5	1.3934 (18)
N1—H6	0.893 (18)	C5—C4	1.380 (2)
C7—C6	1.504 (2)	C5—H5	0.935 (18)
C7—H13	0.93 (2)	C2—C3	1.3917 (18)
C7—H17	0.92 (2)	C3—C4	1.374 (2)
C7—H12	0.96 (2)	C3—H3	0.918 (18)
C8—C2	1.498 (2)	C4—H4	1.00 (2)
C8—H11	0.93 (2)	O1—H2	0.87 (3)
C8—H10	0.92 (2)	O1—H1	0.90 (3)
C8—H9	0.88 (2)		
			
C1—N1—H8	114.1 (10)	C2—C1—N1	118.34 (11)
C1—N1—H7	110.5 (10)	C6—C1—N1	118.50 (11)
H8—N1—H7	106.5 (15)	C1—C6—C5	117.13 (12)
C1—N1—H6	114.0 (11)	C1—C6—C7	121.91 (11)
H8—N1—H6	105.3 (15)	C5—C6—C7	120.95 (12)
H7—N1—H6	105.9 (14)	C4—C5—C6	121.09 (13)
C6—C7—H13	114.5 (12)	C4—C5—H5	121.5 (11)
C6—C7—H17	110.9 (13)	C6—C5—H5	117.4 (11)
H13—C7—H17	103.3 (16)	C1—C2—C3	117.24 (12)
C6—C7—H12	108.9 (13)	C1—C2—C8	122.14 (12)
H13—C7—H12	108.2 (18)	C3—C2—C8	120.62 (12)
H17—C7—H12	111.0 (18)	C4—C3—C2	121.22 (13)
C2—C8—H11	111.7 (12)	C4—C3—H3	119.6 (11)
C2—C8—H10	110.5 (11)	C2—C3—H3	119.2 (11)
H11—C8—H10	109.9 (17)	C3—C4—C5	120.15 (12)
C2—C8—H9	109.7 (13)	C3—C4—H4	118.9 (11)
H11—C8—H9	104.3 (18)	C5—C4—H4	120.9 (11)
H10—C8—H9	110.5 (17)	H2—O1—H1	105 (2)
C2—C1—C6	123.14 (11)		
			
C2—C1—C6—C5	−2.12 (17)	N1—C1—C2—C3	−179.68 (10)
N1—C1—C6—C5	179.69 (10)	C6—C1—C2—C8	−177.60 (12)
C2—C1—C6—C7	176.85 (12)	N1—C1—C2—C8	0.60 (18)
N1—C1—C6—C7	−1.34 (17)	C1—C2—C3—C4	−0.96 (18)
C1—C6—C5—C4	0.94 (17)	C8—C2—C3—C4	178.78 (14)
C7—C6—C5—C4	−178.04 (13)	C2—C3—C4—C5	−0.1 (2)
C6—C1—C2—C3	2.13 (17)	C6—C5—C4—C3	0.1 (2)

### Hydrogen-bond geometry (Å, °)

**Table:**

*D*—H···*A*	*D*—H	H···*A*	*D*···*A*	*D*—H···*A*
O1—H1···Cl1	0.90 (2)	2.41 (2)	3.305 (3)	173 (2)
O1—H2···Cl1^i^	0.87 (3)	2.32 (3)	3.163 (3)	165 (2)
N1—H6···Cl1^ii^	0.893 (18)	2.392 (18)	3.235 (3)	157.5 (15)
N1—H7···O1	0.896 (16)	1.835 (16)	2.731 (3)	177.3 (17)
N1—H8···Cl1^iii^	0.883 (16)	2.414 (16)	3.265 (3)	162.8 (15)

Symmetry codes: (i) *x*, −*y*+1/2, *z*−1/2; (ii) −*x*+1, −*y*, −*z*+1; (iii) *x*, *y*, *z*−1.
